# Evaluation of Aztreonam and Ceftazidime/Avibactam Synergism against *Klebsiella pneumoniae* by MALDI-TOF MS

**DOI:** 10.3390/antibiotics12061063

**Published:** 2023-06-16

**Authors:** Camila Mörschbächer Wilhelm, Everton Inamine, Andreza Francisco Martins, Afonso Luís Barth

**Affiliations:** 1 Programa de Pós-Graduação em Ciências Farmacêuticas, Universidade Federal do Rio Grande do Sul, Porto Alegre 90610-000, Brazil; 2 Laboratório de Pesquisa em Resistência Bacteriana (LABRESIS), Hospital de Clinicas de Porto Alegre, Porto Alegre 90035-903, Brazil; 3 Laboratório Carlos Franco Voegeli, Irmandade Santa Casa de Misericórdia de Porto Alegre, Porto Alegre 90050-170, Brazil

**Keywords:** synergism, MALDI-TOF MS, relative growth, aztreonam, ceftazidime/avibactam

## Abstract

Introduction: Resistance to carbapenems due to the co-production of NDM and ESBL or NDM and KPC is increasing. Therefore, combined therapy with aztreonam (ATM) plus ceftazidime/avibactam (CZA) has been recommended. Then, it is necessary to develop and evaluate fast and simple methods to determine synergism in vitro in microbiology laboratories. Objective: To develop a method to determine the synergism of ATM and CZA by MALDI-TOF MS (SynMALDI). Method: *Klebsiella pneumoniae* (n = 22) isolates with *bla*_NDM_ and/or *bla*_KPC_ genes were tested. The time–kill curve assay was performed for four isolates (three positives for *bla*_NDM_ and *bla*_KPC_ and one positive for *bla*_NDM_ only). For SynMALDI, each isolate was incubated for 3 h in 4 tubes containing brain–heart infusion broth with the following: (1) no antibiotic; (2) ATM at 64 mg/L; (3) CZA at 10/4 mg/L; and (4) ATM at 64 mg/L plus CZA at 10/4 mg/L. After incubation, the bacterial protein extract was analyzed by MALDI-TOF MS, and the relative growth (RG) was determined for each isolate, considering intensities of the peaks of the bacterium incubated with antibiotic (tubes 2, 3, and 4) to the same bacterium incubated without antibiotic (tube 1), as follows: RG = Intensity_With antibiotic_/Intensity_Without antibiotic_. The combination was determined as synergistic when there was an RG decrease of 0.3 in the antibiotic combination in relation to the RG of the most active antibiotic alone. Results: The combination of ATM plus CZA proved to be synergic by time–kill curve assay. All isolates tested with the SynMALDI method also presented synergism. Conclusions: Detection of synergism for ATM plus CZA combination can be determined by MALDI-TOF MS, providing fast results in order to improve patient treatment.

## 1. Introduction

Carbapenem-resistant Enterobacterales (CRE) are a major threat to public health worldwide [1,2]. The treatment of infections caused by CRE can be made by the use of new antibiotic combinations of beta-lactam with beta-lactamase inhibitors, such as ceftazidime/avibactam (CZA). Avibactam is a diazabicyclooctane molecule; it is a beta-lactamase inhibitor that protects ceftazidime from being hydrolyzed by serine beta-lactamases, such as extended-spectrum beta-lactamases (ESBL), *Klebsiella pneumoniae* carbapenemase (KPC) and OXA-48-like. Nonetheless, avibactam is not active against New Delhi Metallo-beta-lactamase (NDM) and other metallo-beta-lactamases (MBL). A therapeutic option for the treatment of infections due to Enterobacterales with NDM carbapenemase is aztreonam (ATM) which, however, is hydrolyzed by serine beta-lactamases [3,4].

The emergence of multidrug-resistant pathogens has been observed around the world [5]. Among Enterobacterales species, *K. pneumoniae* stands out as the main multidrug-resistant pathogen, as its plasmids carrying resistant genes are easily disseminated, especially in hospital environments [6]. Especially, an increase in the spread of genes encoding NDM among Enterobacterales has brought more concern to the problem of antimicrobial resistance, mainly because the production of NDM has also been reported in co-occurrence with KPC and other serine beta-lactamases [7,8,9]. Although new antibiotics, such as imipenem/relebactam, meropenem/vaborbactam, aztreonam/avibactam, and cefiderocol, have demonstrated effectiveness against multidrug-resistant Enterobacterales [6,10], most of them are not currently available in Brazil and other countries. Therefore, treatment options have become very limited when the infection is caused by CRE-co-producing NDM and KPC carbapenemases. Effective treatment for infections due to CRE-co-producing NDM and KPC can be achieved using the combination of CZA plus ATM, as CZA is active against KPC and ATM is active against NDM, whether no other resistance mechanism is associated [4]. The CZA plus ATM-combined therapy has been clinically debated [11,12,13]. Although the Infectious Diseases Society of America (IDSA) and the European Society of Clinical Microbiology and Infectious Diseases (ESCMID) recommend the empiric use of this therapy to treat infections caused by CRE-co-producing NDM and KPC [14,15], it is preferred that the clinical therapy could be guided by synergism experiments in vitro. However, the methodologies for the detection of synergism in vitro are usually labor-intensive and time-consuming [16,17,18,19,20,21].

Several studies of synergism have been described for ATM and CZA: MIC:MIC ratio technique [18,19,21,22,23]; strip crossing [17,21,22]; double-disk [16,19]; combined disk [16]; and several other variations [20,21,24,25,26]. However, only a few have tested and correlated their results to the in vitro standard assay for synergism detection, the time–kill curve (TKC) assay [16,24,25]. As the TKC assay is laborious and time-consuming, it is necessary to develop methods that could be performed in the microbiology laboratory in a practical way. The TKC alternative methods already proposed, although they may be easy to perform, can take up to 48 h to provide the results as they usually require the MIC of the antibiotics to be previously determined. As these methods are based on antimicrobial disk or gradient strip diffusion methods, we understand that a technique that uses the methodology of matrix-assisted laser desorption ionization—time of flight mass spectrometry (MALDI-TOF MS)—can be used to develop a rapid method to evaluate synergism, since MALDI-TOF has already been used to properly provide antimicrobial susceptibility profile [27].

MALDI-TOF MS has revolutionized the microbiology field regarding microbial identification. Furthermore, this methodology has been used for other applications such as (1) the detection of beta-lactamase activity through hydrolysis assays, (2) the direct detection of beta-lactamase enzymes, and (3) the determination of antimicrobial susceptibility by the evaluation of the relative growth rate [28]. The latter was developed by Lange et al. (2014) and named MALDI Biotyper-antimicrobial susceptibility testing rapid assay (MBT-ASTRA). The MBT-ASTRA compares the spectrum of the bacterium incubated with antibiotic with the spectrum of the same bacterium incubated without antibiotic [1,2]. This methodology has demonstrated satisfactory results for meropenem, ceftazidime, ciprofloxacin, and other antibiotics against mainly Enterobacterales [27,29,30,31].

Therefore, the objective of this study was to develop and evaluate a rapid methodology, termed SynMALDI, to detect ATM plus CZA synergism by MALDI-TOF MS.

## 2. Materials and Methods

### 2.1. Bacterial Strains

A total of 22 *Klebsiella pneumoniae* clinical isolates, obtained from 2019 to 2022, stored at the bacterial bank of Laboratório de Pesquisa em Resistência Bacteriana (LABRESIS) were selected by convenience and included in the study. All isolates were obtained from a hospital in the city of Porto Alegre, Brazil, and were isolated from the urinary tract, rectal swab, and wound. Identification was performed with MALDI-TOF MS (Bruker Daltonics^®^, Bremen, Germany) according to the manufacturer’s instructions. For 21 meropenem-resistant isolates (Kp01 to Kp21), the presence of the carbapenemase genes was previously evaluated by high-resolution melting real-time PCR (qPCR-HRM), using a multiplex assay with primers for *bla*_KPC_, *bla*_NDM_, *bla*_OXA-48-like_, *bla*_IMP_, *bla*_GES_, and *bla*_VIM_ genes, as previously described [32]. One meropenem susceptible isolate (Kp22) was included as a negative control of the SynMALDI method.

**Figure 1:**
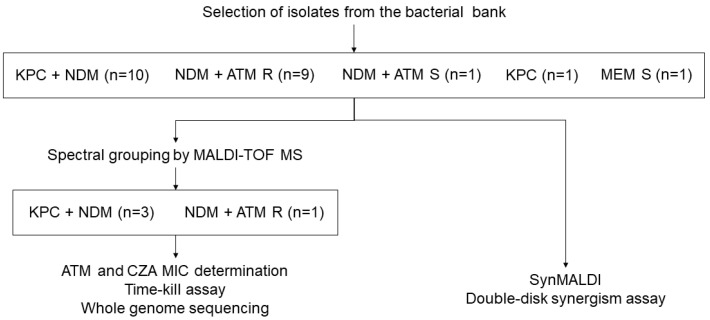
Flow chart demonstrating the procedures performed in the study. ATM: aztreonam. CZA: ceftazidime/avibactam. MEM: Meropenem. MIC: Minimum inhibitory concentration. R: Resistant. S: Susceptible.

In order to avoid testing isolates closely related in the TKC, we performed spectral grouping with data acquired by MALDI-TOF MS using the same parameters for microbial identification. Spectra were grouped using ClinProTools 3.0 (Bruker Daltonics^®^), as described elsewhere [33], resulting in eight groups (A–H). One NDM producer and ATM resistant and three NDM and KPC co-producers from distant spectral groups were selected for TKC experiments and for whole genome sequencing (WGS) in order to identify beta-lactamase genes (Figure 1).

### 2.2. Determination of Susceptibility by Disk Diffusion and Minimum Inhibitory Concentration (MIC)

Antimicrobial susceptibility testing by disk diffusion and gradient diffusion strips was performed for ATM and CZA, with disks (Oxoid Thermo Scientific^®^, Waltham, MA, USA) of 30 µg and 10/4 µg, respectively. The results were interpreted according to EUCAST [34]. Disk diffusion and MIC breakpoints for ATM and CZA are displayed in Table 1. Quality control was performed with *Escherichia coli* ATCC 25922, according to EUCAST instructions [35]. Antimicrobial susceptibility testing by disk diffusion was performed for all isolates, and MIC was determined by gradient diffusion strip for the isolates selected for TKC assay.

### 2.3. Time–Kill Curve (TKC) Assay

TKC assay was performed according to CLSI guidelines [36] with some modifications, as previously described [37]. Briefly, fresh cultures grown on blood agar plates were used to prepare an inoculum of approximately 3 × 10^8^ CFU/mL in cation-adjusted Müeller–Hinton (CAMH) broth. This suspension, in the log phase, was diluted 1:5 and added to four tubes containing CAMH to achieve a final concentration of 6 × 10^5^ CFU/mL. The first tube was without antibiotic; the second tube contained ATM at 8 mg/L (BioChimico^®^, Rio de Janeiro, Brazil); the third tube contained CZA at 8/2 mg/L (ceftazidime at 8 mg/L and avibactam at 2 mg/L; Wyeth/Pfizer^®^, São Paulo, Brazil), and the fourth tube contained ATM at 8 mg/L and CZA at 8/2 mg/L. All experiments were performed in duplicates. The tubes were incubated at 37 °C, at 125 rpm agitation for 24 h. An aliquot from each tube was removed at times 0, 1, 2, 4, 6, 12, and 24 h and serially (1:10) diluted. A volume of 20 µL of each dilution from the same tube was spotted on a MacConkey agar plate. Plates were incubated at 37 °C for 18 to 24 h. Colonies were counted considering the spots with 10 to 100 colonies, and the results were reported as CFU/mL. Synergism was defined as a decrease ≥ 2 log_10_ CFU/mL by the antibiotic combination compared to the most active antibiotic alone, and antagonism was defined as an increase ≥ 2 log_10_ CFU/mL by the combination compared with the most active antibiotic alone after 24 h of incubation [36].

### 2.4. Whole Genome Sequencing (WGS)

Genomic DNA was extracted from colonies grown on LB broth using a QIAamp DNA Mini Extraction Kit (QIAGEN^®^, Hilden, Germany). Total DNA concentration was measured using a Qubit dsDNA HS Assay Kit with a Qubit 4 fluorometer (Thermo Fisher Scientific). WGS was performed using the Illumina MiSeq™ platform (Illumina, San Diego, CA, USA). The paired-end library was constructed with the NexteraTM XT DNA Library Prep Kit (MiSeq™ System), and the MiSeqTM Reagent V2 kit (2 × 250 cycles) was used to perform a run with a coverage depth of 100×. The raw reads were quality-trimmed (Q > 30) and assembled using CLC Genomic Workbench 21. Antimicrobial resistance genes were identified (contigs > 200 bp) in silico using ResFinder 4.0 (https://cge.food.dtu.dk/services/ResFinder/, accessed on 26 December 2022) and QIAGEN Microbial Insight-Antimicrobial Resistance database (QMI-AR).

### 2.5. Antibiotic Solution Preparation for Synergism by MALDI-TOF MS (SynMALDI)

For the synergism determination by MALDI-TOF MS, antibiotic solutions were prepared from commercially available disks of ATM 30 µg and CZA 10/4 µg (Oxoid Thermo Scientific^®^). Each antibiotic lot of disks was quality-control-checked, as recommended by EUCAST [35]. The disks were eluted for 15 min in distilled water in order to obtain ATM at 64 mg/L and CZA at 10/4 mg/L as final concentrations.

### 2.6. Synergism by MALDI-TOF MS (SynMALDI)

The method for determination of synergism by MALDI-TOF MS was adapted from MBT-ASTRA [27] and performed for all isolates. Isolates were suspended in brain–heart infusion broth (Kasvi, São José dos Pinhas, Brazil) to obtain turbidity equivalent to 0.5 McFarland. In order to reach a final volume of 200 µL, 50 µL of the bacterial suspension was added to four microtubes as follows: the first without antibiotic; the second tube with ATM; the third tube with CZA; and the fourth tube with ATM and CZA. The microtubes were vortexed and incubated, without agitation, for 3 h at 35 ± 2 °C. Afterward, the suspensions were centrifuged at 16,060× *g* (13,000 rpm) for 2 min. The supernatant was discarded, and 150 µL of distilled water was added to the microtubes. The microtubes were vortexed and centrifuged at 16,060× *g* (13,000 rpm) for 2 min. The supernatant was discarded, and 100 µL of ethanol 70% (Merck^®^, Darmstadt, Germany) was added to each microtube. The microtubes were vortexed, left to rest at room temperature for 5 min, and centrifuged at 16,060× *g* (13,000 rpm) for 2 min. The supernatant was discarded, and the pellet was left to air dry at room temperature. Subsequently, 10 µL of formic acid 70% (Sigma–Aldrich^®^/Merck^®^, Darmstadt, Germany) and 10 µL of acetonitrile 100% (Merck^®^) containing an internal standard (RNase B 20 g/L; Sigma–Aldrich^®^) were added, and the microtubes were vortexed and centrifuged at 16,060× *g* (13,000 rpm) for 2 min. A volume of 1 µL of the supernatant was spotted, in quadruplicates, onto a polished steel target plate and left to air dry. A volume of 1 µL of α-cyano-4-hidroxy-cinnamic acid (HCCA; Bruker) at 10 mg/mL in acetonitrile 50% and trifluoracetic acid 2.5% (Sigma–Aldrich^®^) was added, and the spots were left to air dry at room temperature.

For spectra acquisition, a Microflex LT (Bruker Daltonics^®^) mass spectrometer was used with flexControl 3.4 software (Bruker Daltonics^®^). The parameters applied were the same as used for microbial identification (ion source 1, 20 kV; ion source 2, 18.25 kV; lens, 6 kV; detector gain, 2850 V), with a range from 2.000 to 20.000 Da (method MBT_FC.par, set by the manufacturer). The mass spectrometer was externally calibrated using the bacterial test standard—BTS (Bruker^®^, Billerica, MA, USA), according to manufacturer instructions. An automated MBT_AutoX programming was used for the acquisition of the spectra.

Spectra were analyzed with flexAnalysis 3.4 software (Bruker Daltonics^®^). For each spectrum, four peaks were manually selected (two peaks from the bacterium and two peaks from the internal standard), and their intensities were annotated after baseline subtraction and smoothing. Peaks from bacteria were selected between the ranges 6000–6300 and 9000–10,000 *m*/*z*, while peaks from the internal standard were approximately 7454 and 14,910 *m*/*z*. The peaks from the bacteria were carefully selected after comparison with the spectrum of RNase B alone in order to avoid choosing peaks from the internal standard. For each quadruplicate, the sum of the intensities from the bacterium (Int_Bac_) was normalized with the sum of the intensities from the internal standard (Int_RNase B_). Then, the RG value was calculated by the ratio of the median of the normalized peaks from bacteria incubated with antibiotic (Int_ATB_) with the median of the normalized peaks from bacteria incubated without antibiotic (Int_BHI_). RG values were calculated as follows: RG = Median (∑ Int_Bac+ATB_/∑ Int_RNase B+ATB_)/Median (∑ Int_Bac+BHI_/∑ Int_RNase B+BHI_). For each isolate, three RG values were calculated, considering ATM and CZA alone and in combination: RG ATM; RG CZA; and RG ATM + CZA. Synergism was defined as a decrease ≥ 0.3 at the RG value of the antibiotic combination compared to the RG value of the most active antibiotic alone, whether RG ATM or RG CZA.

### 2.7. Double-Disk Synergism Assay

The double-disk method to evaluate synergism was performed according to EUCAST standard susceptibility testing by disk diffusion method [38], with ATM 30 µg and CZA 10/4 µg disks placed at a 20 mm center-to-center distance. Such distance was chosen because it is the same distance between disks when using an antibiotic disk dispenser device. When the presence of an inhibition zone, also known as a “ghost zone”, was observed between the disks, the test was considered positive for synergism. Double-disk synergism testing was performed for all isolates except for Kp20 to Kp22.

## 3. Results

**Figure 2:**
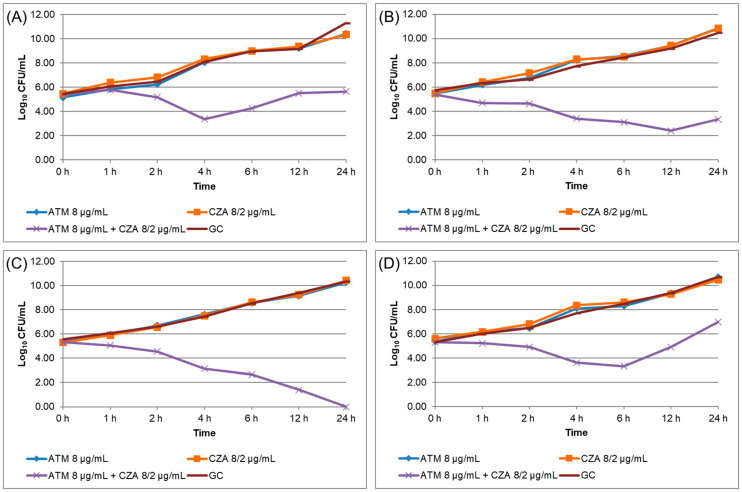
Time–kill curve assay results performed for isolates Kp04 (**A**), Kp08 (**B**), Kp10 (**C**), and Kp16 (**D**). ATM: aztreonam. CZA: ceftazidime/avibactam.

A total of 19 *K. pneumoniae* isolates (Kp01 to Kp19) were resistant to both ATM and CZA by disk diffusion standard susceptibility testing, presenting *bla*_NDM_ and/or *bla*_KPC_ genes as determined by qPCR-HRM; all isolates were analyzed by MALDI-TOF-MS for spectral grouping (Table 2). The four isolates (Kp04, Kp08, Kp10, and Kp16) that were tested for TKC assay presented high MIC for ATM and CZA, and all were positive for synergism (Table 3), as the combination of ATM and CZA presented a decrease ≥ 2 log_10_ CFU/mL in comparison to each antibiotic alone (Figure 2). WGS of these four isolates confirmed the presence of carbapenemase genes and demonstrated the presence of ESBL genes (Table 3).

**Figure 3:**
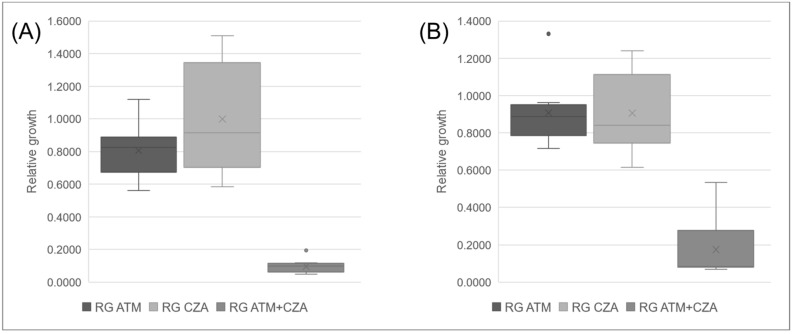
Boxplot of mean values of relative growth (RG) for aztreonam (ATM) and ceftazidime/avibactam (CZA) alone and in combination. (**A**) ATM and CZA resistant isolates positive for *bla*_KPC_ and *bla*_NDM_. (**B**) ATM and CZA-resistant isolates positive for *bla*_NDM_ only.

The isolates tested for TKC were tested for SynMALDI and demonstrated a significant decrease at RG of ATM plus CZA in comparison to RG of ATM and of CZA individually. Subsequently, all isolates were tested for SynMALDI (Table 4). ATM-resistant isolates presented RG of ATM ≥ 0.5617 and a mean ± standard deviation of 0.8520 ± 0.1655, while CZA-resistant isolates presented RG of CZA ≥ 0.5841 and a mean of 0.9341 ± 0.2715 (Figure 3). The RG of ATM plus CZA ranged from 0.0486 to 0.5338, with a mean of 0.1335 ± 0.1293. The comparison of the RG of ATM plus CZA with individual results of RG of ATM and CZA indicated that all isolates presented a lower value when both antibiotics were combined, with a minimum difference to RG ATM of 0.3630 and to RG CZA of 0.4709. Therefore, we considered that all isolates, resistant to both ATM and CZA, demonstrated synergism by SynMALDI, as all presented a minimum difference of 0.3 of RG of ATM plus CZA values in relation to the RG of the most active antibiotic alone. Interestingly, the mean of the RG differences considering ATM and CZA, in comparison to ATM plus CZA, were 0.7186 ± 0.1647 and 0.8155 ± 0.2817, respectively. Isolates tested as control (Kp20, Kp21, and Kp22) that were susceptible to ATM and/or CZA demonstrated RG values ≤ 0.3426 and ≤ 0.0647, respectively. These three isolates did not present a minimum difference of 0.3 of the RG of ATM plus CZA compared to the RG of the single most active antibiotic.

Double-disk synergism, which was performed for isolates resistant to both ATM and CZA, demonstrated the “ghost zone”, indicating a positive result of synergism for all isolates (Kp01 to Kp19).

## 4. Discussion

Although clinical studies demonstrating the efficacy of ATM plus CZA to treat infections of CRE co-producing NDM and KPC are scarce, empiric treatment with this combination of antibiotics is recommended by IDSA and ESCMID [14,15]. However, the synergism of ATM plus CZA is expected if the resistance mechanism is due to serine and MBL co-production only. Resistance to CZA by mechanisms other than MBL production has been reported, such as amino acid substitutions at the active site of beta-lactamases (which is the case of isolates harboring KPC-3 mutations), decreased membrane permeability due to porin and/or PBP mutations, among others [39]. Synergism of ATM plus CZA may not occur if resistance to CZA is due to resistant mechanisms other than MBL production, and in order to better guide the therapy, it is necessary to evaluate in vitro whether ATM and CZA are synergic. Thus, we developed a fast and easy method to determine ATM and CZA synergism with the use of MALDI-TOF MS.

The principle of the TKC assay to evaluate synergism is based on the decrease in the growth rate of the bacterium incubated with combined antibiotics when compared to the most active agent alone. We have used the same principle to consider synergism by the SynMALDI method, i.e., a significant decrease in the RG value of the bacterium with combined antibiotics versus the bacterium with the antibiotic alone. In our study, for the determination of ATM and CZA synergism, we found a minimal difference of around 0.3 to 0.4 and a mean difference of around 0.7 to 0.8. Considering a decrease ≥ 0.3 of RG value of ATM plus CZA, all isolates that were resistant to both ATM and CZA were considered positive for synergism by SynMALDI, as was expected according to TKC and double-disk synergism assays. Conversely, it was also expected that control strains Kp20 to Kp22 would not present synergism since they already presented susceptibility and low RG values for ATM or CZA individually; therefore, the addition of a second antibiotic would not significantly increase the activity of the first antibiotic against the bacterium. Therefore, we suggest performing SynMALDI for ATM with CZA only if the isolate demonstrates to be resistant to both antibiotics individually.

Studies have assessed ATM plus CZA combination by several in vitro methods, attempting to propose a way to detect synergism. Among methodologies using gradient strips, MIC:MIC ratio is one of the most used. Although some studies [18,22] have calculated the fractional inhibitory index, a measure usually determined using the checkerboard assay to indicate synergy, the MIC:MIC ratio technique is laborious, expensive, and may be subjective as, depending on the MIC, an inhibition zone around all strip can be formed [18]. Similar methods that have been used are the direct overlaid of the strips [19] and a disk placed next to the strip [40], which present the same difficulties for interpretation as MIC:MIC ratio method. Another method also used to evaluate ATM plus CZA synergism was strip crossing [17,21,22,41]. All methods using gradient strips require the MIC to be previously determined, which means that it will increase by at least one more day to provide synergy results. Moreover, such methods will present increased costs, as gradient strips are expensive. As an alternative, a double-disk synergy test can be performed in order to reduce costs, although it is a qualitative assay and it requires an overnight incubation [42].

Regardless of the fact that there are several methods to evaluate synergism in vitro, the standard assays are only TKC and checkerboard. Regarding ATM and CZA combination, few studies have performed TKC [16,24,25,43] and checkerboard [21,43,44]. They all have found synergism for the ATM plus CZA combination; however, both assays are laborious and time-consuming, which would not be suitable for a routine microbiology laboratory. The studies mentioned above assessing ATM plus CZA synergism have tested various isolates regarding beta-lactamase production, such as KPC, NDM, other MBL, OXA, and/or ESBL producers; however, only a few isolates were carbapenemase co-producers, such as NDM and OXA [43,44]. In fact, two studies evaluated isolates that were KPC and NDM co-producers [23,43]. We aimed to test carbapenemases-producing isolates according to our local epidemiology, which indicates an increase in NDM dissemination with the co-occurrence of NDM and KPC [9]. Similar to our results, studies that tested NDM and ESBL producers by TKC also found synergism of ATM plus CZA combination [16,24,25]. The first study that evaluated one isolate co-producing KPC and NDM performed a strip crossing method, also demonstrating synergism [23]. On the other hand, Lu et al. (2022) demonstrated synergistic activity of ATM plus CZA combination by TKC assays for one Enterobacter cloacae IMP-producer and one *Escherichia coli* NDM-producer that were resistant to both antibiotics. Additionally, they showed synergism by checkerboard for other species co-producing KPC and NDM, including *K. pneumoniae*, by checkerboard method [43]. A limitation of our study and others is that isolates expressing KPC variants that led to CZA resistance, such as KPC-35, KPC-78, and KPC-33 [45], were not tested to assess whether synergistic effects would still occur. Although it was not possible to correlate with clinical outcomes, our results by SynMALDI were confirmed by TKC, which was performed for four representative isolates of our sample, all *K. pneumoniae*, and by the double-disk synergism method.

The method we presented here, SynMALDI, could be performed and provide synergy results in approximately 4 to 5 h, and once the laboratory already has a MALDI-TOF mass spectrometer, the costs would be very low (less than US$ 2.00). Additionally, it is a simple method to be performed, and it does not require additional software for the interpretation of results. Another advantage is that this methodology can presumably be adapted for other antibiotic combinations. We adjusted conditions for CZA to correspond to the same conditions of ATM in order to perform the assay for these antibiotics combined. This is, so far, the first method proposed to detect synergism by MALDI-TOF MS.

## 5. Conclusions

As resistance mechanisms against beta-lactam antibiotics tend to evolve faster than new antibiotics are developed, combined therapy with two or more antibiotics is recommended. Therefore, in order to help guide patient therapy for infections due to CRE co-producers of carbapenemase, it is important to develop fast methods, such as SynMALDI, for synergism detection that can be performed in microbiology routine laboratories.

## Figures and Tables

**Table 1:** Aztreonam and ceftazidime/avibactam breakpoints for disk diffusion and minimal inhibitory concentration, according to EUCAST.

Antibiotic	Disk Diffusion (mm)		MIC (mg/L)	
	S	R	S	R
Aztreonam	≥26	<21	≤1	>4
Ceftazidime/avibactam	≥13	<13	≤8	>8

S: Susceptible, standard dosing regimen. R: Resistant.

**Table 2:** Carbapenemases, spectral grouping, and susceptibility of all isolates included in this study.

Isolate	Cp	Cluster	ATM		CZA	
			DD (mm)	Cat.	DD (mm)	Cat.
Kp01	KPC, NDM	F	8	R	7	R
Kp02	KPC, NDM	H	10	R	6	R
Kp03	KPC, NDM	A	6	R	8	R
Kp04	KPC, NDM	A	6	R	8	R
Kp05	KPC, NDM	H	6	R	6	R
Kp06	KPC, NDM	E	6	R	9	R
Kp07	KPC, NDM	C	6	R	6	R
Kp08	KPC, NDM	E	6	R	8	R
Kp09	KPC, NDM	E	6	R	9	R
Kp10	KPC, NDM	G	6	R	8	R
Kp11	NDM	G	6	R	9	R
Kp12	NDM	G	12	R	7	R
Kp13	NDM	B	8	R	8	R
Kp14	NDM	G	9	R	10	R
Kp15	NDM	D	6	R	6	R
Kp16	NDM	F	6	R	6	R
Kp17	NDM	F	6	R	6	R
Kp18	NDM	C	6	R	6	R
Kp19	NDM	D	6	R	8	R
Kp20	NDM	D	30	S	8	R
Kp21	KPC	F	6	R	20	S
Kp22	NT	F	30	S	24	S

ATM: Aztreonam. Cat.: Antimicrobial susceptibility categorization according to EUCAST. Cp: Carbapenemase. CZA: Ceftazidime/avibactam. DD: disk diffusion. R: Resistant. S: Susceptible. NT: Not tested.

**Table 3:** Minimum inhibitory concentration and time–kill curve assay results of four representative isolates.

Isolate	Beta-Lactamase Genes	ATM		CZA		ATM + CZA
		MIC (mg/L)	TKC *	MIC (mg/L)	TKC *	TKC *
Kp04	NDM-1, KPC-2, CTX-M-15, SHV-187, TEM-181	>1024	10.42	>256	10.34	5.65
Kp08	NDM-1, KPC-2, OXA-1, SHV-187, TEM-181	>1024	10.86	>256	10.83	3.34
Kp10	NDM-1, KPC-2, OXA-1, CTX-M-15, SHV-187, TEM-181	>1024	10.28	>256	10.42	0.00
Kp16	NDM-1, OXA-1, CTX-M-15, SHV-187, TEM-181	>1024	10.70	>256	10.46	6.98

* Log_10_ CFU/mL at 24 h incubation in time–kill curve (TCK) assay. ATM: Aztreonam. CZA: Ceftazidime/avibactam. MIC: Minimum inhibitory concentration.

**Table 4:** Relative growth (RG) values obtained for aztreonam (ATM) and ceftazidime (CZA) in combination and alone.

Isolate	ATM + CZA	ATM		CZA	
	RG	RG	Dif. *	RG	Dif. *
Kp01	0.1082	1.1196	1.0114	1.4997	1.3915
Kp02	0.0544	0.8271	0.7726	0.9160	0.8616
Kp03	0.1190	0.7915	0.6725	1.1907	1.0717
Kp04	0.0793	0.9360	0.8566	0.9123	0.8330
Kp05	0.1938	0.5617	0.3679	0.6647	0.4709
Kp06	0.0704	0.7019	0.6316	0.7411	0.6708
Kp07	0.0966	0.8282	0.7315	0.9710	0.8744
Kp08	0.1042	0.8389	0.7346	1.5111	1.4068
Kp09	0.0486	0.6440	0.5955	0.5841	0.5356
Kp10	0.0841	0.8880	0.8040	0.7833	0.6993
Kp11	0.0874	0.8129	0.7255	0.6141	0.5267
Kp12	0.1127	0.7160	0.6033	0.7209	0.6082
Kp13	0.4411	1.3313	0.8902	0.9592	0.5181
Kp14	0.0912	0.7752	0.6840	0.8864	0.7952
Kp15	0.0821	0.7618	0.6797	0.7712	0.6891
Kp16	0.0696	0.9395	0.8699	1.0962	1.0266
Kp17	0.0796	0.9623	0.8827	0.8398	0.7602
Kp18	0.0813	0.8581	0.7768	1.2394	1.1581
Kp19	0.5338	0.8967	0.3630	1.1305	0.5967
Kp20	0.0528	0.3416	0.2888	0.6501	0.5973
Kp21	0.0585	0.8503	0.7918	0.0647	0.0062
Kp22	0.0311	0.2195	0.1884	0.0314	0.0003

* Difference of RG value in relation to RG ATM + CZA.

## Data Availability

Data is contained within the article and are available from the corresponding author upon reasonable request.
